# P-612. Is Respiratory Gram Stain Useful in the Era of Rapid, Highly Sensitive Molecular Pneumonia Panels?

**DOI:** 10.1093/ofid/ofaf695.825

**Published:** 2026-01-11

**Authors:** Calvin Albrecht, Jonathan H Ryder, Elizabeth Lyden, Matt Anderson, Trevor C Van Schooneveld

**Affiliations:** University of Nebraska Medical Center, Omaha, NE; University of Nebraska Medical Center, Omaha, NE; University of Nebraska Medical Center, Omaha, NE; UNMC College of Public Health, Omaha, Nebraska; University of Nebraska Medical Center, Omaha, NE

## Abstract

**Background:**

Historically, respiratory tract Gram stain (GS) has provided rapid results. A randomized trial showed GS can guide empiric antibiotics and facilitate de-escalation. The emergence of the BioFire pneumonia panel (PNP), a multiplex PCR test, calls the utility of GS into question. Our goal is to assess the role of GS in the era of highly sensitive molecular testing.

**Figure:**
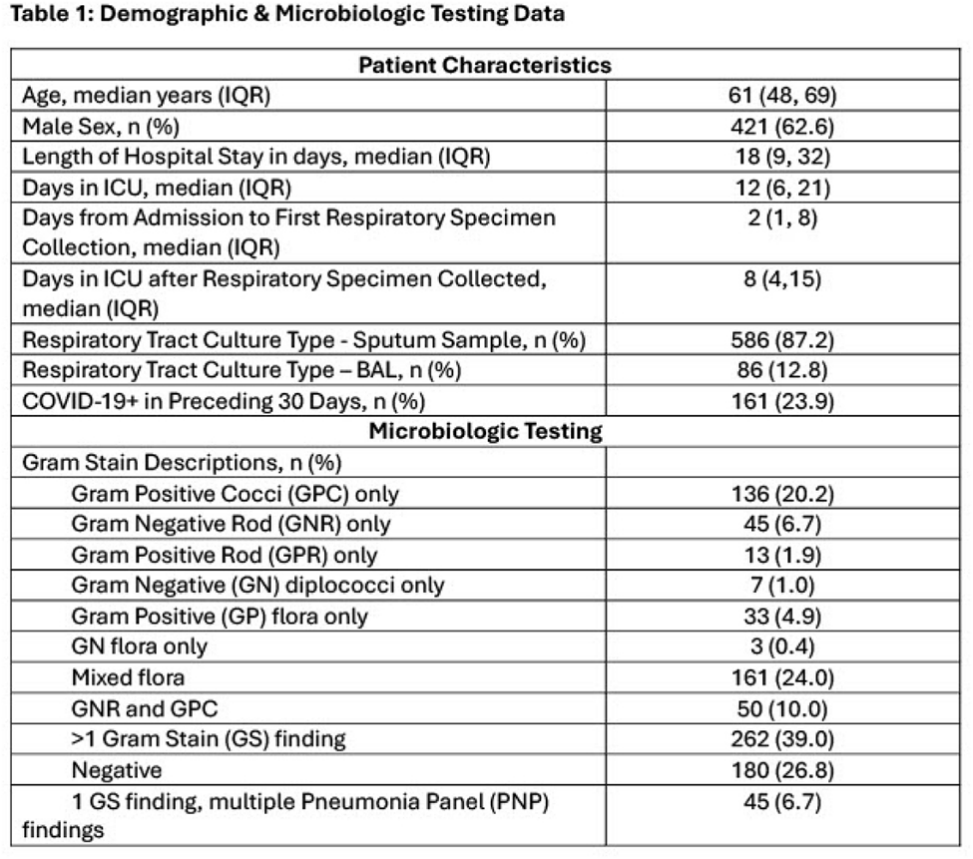

**Figure:**
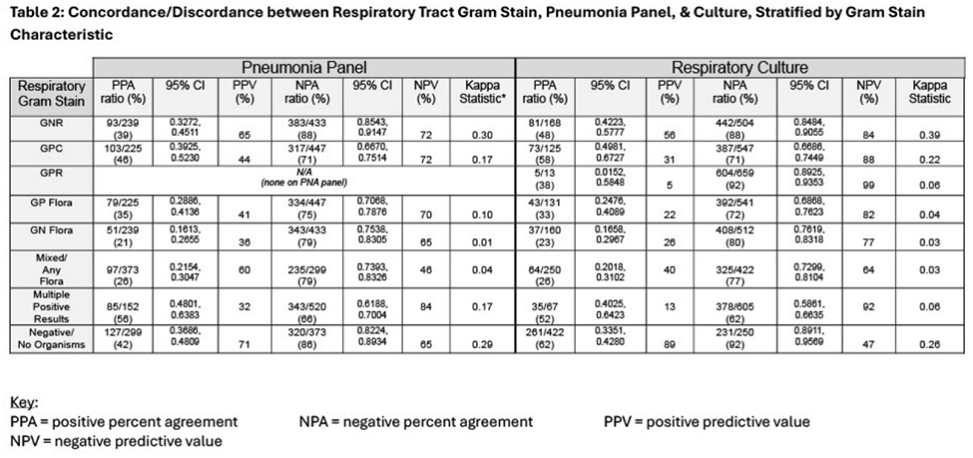

**Methods:**

Respiratory tract specimens from ICU patients who underwent PNP testing at our academic hospital from 02/2021-01/2022 were evaluated for concordance among PNP, GS, and culture (Cx). Positive and negative percent agreement (PPA and NPA) were calculated and compared to the “gold standards” of PNP & Cx. Results were stratified by GS result. PPA = number of positive results on both GS and gold standard (PNP or Cx) divided by number of positive results on gold standard. NPA = number of negative results on both GS and gold standard divided by number of negative results on gold standard. Positive and negative predictive values and kappa statistic were calculated.

**Figure:**
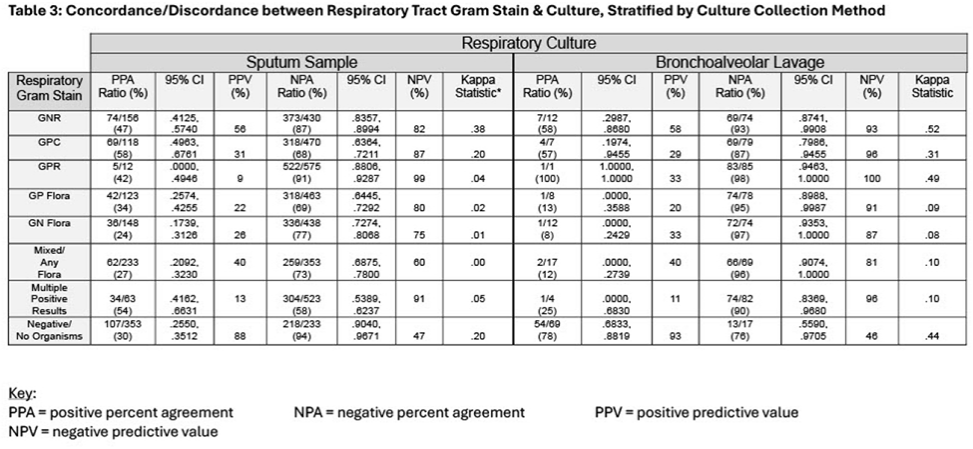

**Figure:**
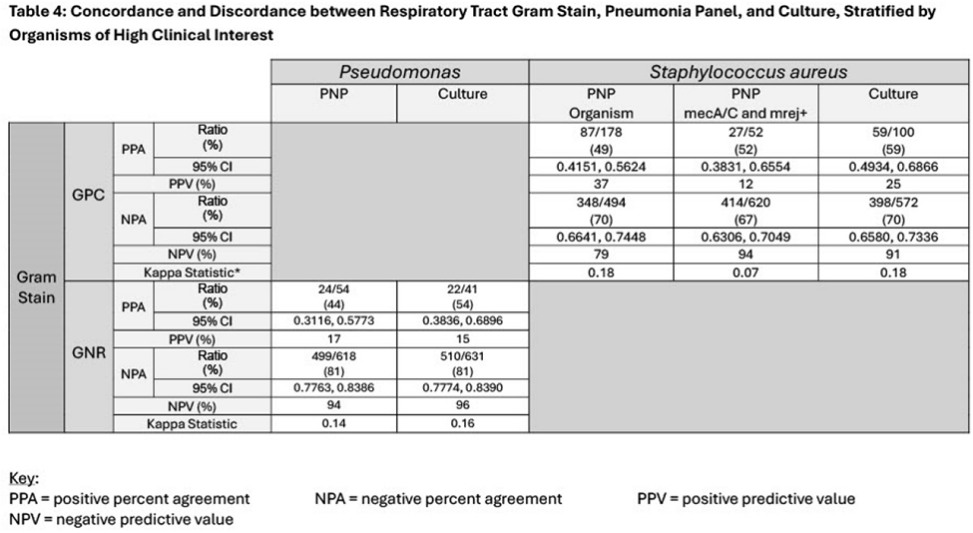

**Results:**

Patient demographics are shown in Table 1. Table 2 shows concordance among the 3 microbiologic test modalities (GS, PNP, Cx). PPA and PPV were low/moderate across all Gram stain types (none were higher than 60% aside from negative Gram stain). NPA and NPV were higher, especially for Gram positive organisms (70-90% range). Negative GS has high NPA and PPV compared to PNP (86%, 71%) and Cx (92%, 89%). Table 3 stratifies Cx comparisons by BAL vs sputum source. Table 4 shows concordance and discordance of GS, PNP, and Cx with regard to highly clinically relevant pathogens (*Pseudomonas, Staphylococcus aureus*), for which PPA (40-50%) and PPV (10-30%) were moderate; NPA (70-80%) and NPV (80-90%) were high.

**Conclusion:**

Respiratory tract GS results have poor concordance with PNP and Cx, as shown by low kappa statistics. BAL GS has higher NPV and NPA than sputum, and GS has high NPA and NPV (especially for *MRSA* and *Pseudomonas*). GS has limited PPA and PPV with PNP or Cx. Consistent with prior studies, our conclusion is that GS alone is useful in ruling out resistant pathogens such as *MRSA* and *Pseudomonas* and when combined with PNP increases confidence in therapy de-escalation. Next steps include evaluating the performance of combined GS and PNP in ruling out the need for *MRSA* and *Pseudomonas* therapy.

**Disclosures:**

Trevor C. Van Schooneveld, MD, FSHEA, FIDSA, BioMerieux: Advisor/Consultant|BioMerieux: Grant/Research Support

